# MED29/434: Using C&IT for Continuing Medical Education in Africa - a pilot project in Zimbabwe

**DOI:** 10.2196/jmir.1.suppl1.e67

**Published:** 1999-09-19

**Authors:** G Robinson, M Dobson, J Sewell

**Affiliations:** ^1^University of Oxford, OxfordUK

**Keywords:** Medical, Education, Education, Distance, Anaesthesia, Electronic Mail, Multimedia

## Abstract

**Introduction:**

There are many problems associated with continuing medical education in developing countries. For clinicians in district hospitals the difficulties can be extreme, including having little access to relevant literature or contact with colleagues. "World Anaesthesia" addressed these problems in two key areas:1. to improve the level of anaesthetic training among non-specialists working in district hospitals2. the introduction and exposure to appropriate anaesthetic technology, capable of being understood, used, serviced and sustained at local level WA produces an educational journal "Update in Anaesthesia", targeted at anaesthetists in developing countries. It has been widely acclaimed by its recipients, who receive it free of charge. "Update" is a recognised teaching resource, and there have been requests for reprints. This is not viable, however the journals were readily converted to hypertext and Adobe PDF and are available from the WFSA web site (http://www.nda.ox.ac.uk/wfsa/). Permission has been obtained from anaesthetic journals, to reproduce review articles. The reviews are more than 2 years old and can be a priceless resource to those without access to a good library.

**Methods:**

Zimbabwe has a level of development and infrastructure that enabled a feasibility study to be carried out on a distance learning project based on WA resources. There is little access to the WWW, except for those in urban areas with sufficient funds to be able to afford an ISP. The WA/WFSA web site and journal articles were put on a CD-ROM and a feasibility study was made to Zimbabwe in April 1999. A range of hospitals were visited, some having received well specified IBM PCs and printers from DANEDA. This gave the opportunity to observe potential recipients at first hand. Some of whom were first time users of a GUI environment.

**Results:**

It was obvious that the materials were being provided in an acceptable format and were of great relevance. Hypertext format is more flexible, as it enable the user to both read on screen and produce hardcopy. PDF documents provides some consistency in hardcopy but the multiple columns makes them harder to read on screen.

**Discussion:**

Lack of relevant clinical training materials is a major obstacle to improving health standards in many developing countries. CD-ROM's offer a cheap way of distributing resource and reference material. It is now possible to provide an "electronic library" covering a range of medical disciplines, for hospitals previously denied these resources. E-mail provides an opportunity to provide the essential personal contact with a tutor. The successful implementation of a distance learning project requires the integration of a number of strands, including clinical, technical and professional training and the creation of suitable systems. It also requires flexibility in order for it to be implemented, dependent of the available facilities and infrastructure in the target region.

